# Andrographolide inhibits arrhythmias and is cardioprotective in rabbits

**DOI:** 10.18632/oncotarget.18051

**Published:** 2017-05-22

**Authors:** Mengliu Zeng, Wanzhen Jiang, Youjia Tian, Jie Hao, Zhenzhen Cao, Zhipei Liu, Chen Fu, Peihua Zhang, Jihua Ma

**Affiliations:** ^1^ Cardio-Electrophysiological Research Laboratory, Medical College of Wuhan University of Science and Technology, Wuhan, Hubei, China

**Keywords:** andrographolide, sodium current, L-type calcium current, action potential, arrhythmia

## Abstract

Andrographolide has a protective effect on the cardiovascular system. To study its cardic-electrophysiological effects, action potentials and voltage-gated Na^+^ (I_Na_), Ca^2+^ (I_CaL_), and K^+^ (I_K1_, I_Kr_, I_to_ and I_Kur_) currents were recorded using whole-cell patch clamp and current clamp techniques. Additionally, the effects of andrographolide on aconitine-induced arrhythmias were assessed on electrocardiograms *in vivo*. We found that andrographolide shortened action potential duration and reduced maximum upstroke velocity in rabbit left ventricular and left atrial myocytes. Andrographolide attenuated rate-dependence of action potential duration, and reduced or abolished delayed afterdepolarizations and triggered activities induced by isoproterenol (1 μM) and high calcium ([Ca^2+^]_o_=3.6 mM) in left ventricular myocytes. Andrographolide also concentration-dependently inhibited I_Na_ and I_CaL_, but had no effect on I_to_, I_Kur_, I_K1_, or I_Kr_ in rabbit left ventricular and left atrial myocytes. Andrographolide treatment increased the time and dosage thresholds of aconitine-induced arrhythmias, and reduced arrhythmia incidence and mortality in rabbits. Our results indicate that andrographolide inhibits cellular arrhythmias (delayed afterdepolarizations and triggered activities) and aconitine-induced arrhythmias *in vivo*, and these effects result from I_Na_ and I_CaL_ inhibition. Andrographolide may be useful as a class I and IV antiarrhythmic therapeutic.

## INTRODUCTION

The traditional medical herb, *Andrographis paniculata* (Burm.f.) Wall. ex Nees, has been used for centuries to treat a variety of diseases in China. Andrographolide, its major bioactive component, has many pharmacological effects, including antitumor [1, 2], anti-inflammatory [1–4], antiviral [2], anti-hypertension [5], antioxidant [3, 6, 7], antihyperglycaemic [2], hepatoprotective [2, 7], and cardioprotective properties [5–11]. Recent studies found that andrographolide protected rat cardiomyocytes against hypoxia/reoxygenation injury by upregulating cellular-reduced glutathione levels and antioxidant enzyme activities [6]. Andrographolide protected mouse hearts from LPS-induced cardiac malfunctions, and thus may prevent myocardial malfunction during sepsis [8]. Because platelet activation is closely linked to coronary heart disease, andrographolide could be used to treat patients with thromboembolic disorders due to its antiplatelet activity [9–11].

Andrographolide has been used clinically for many years, and its cardioprotective properties are known. However, its effects on sodium (I_Na_), calcium (I_CaL_), and potassium channels (I_K1_, I_Kr_, I_to_, I_Kur_), and action potentials (APs) in cardiomyocytes have not been reported. This study explored the effects of andrographolide on the aforementioned ion channels and action potentials (APs) to determine its antiarrhythmic mechanism of action.

## RESULTS

### Effects of andrographolide on APs in rabbit left ventricular and left atrial myocytes

**Figure 1:**
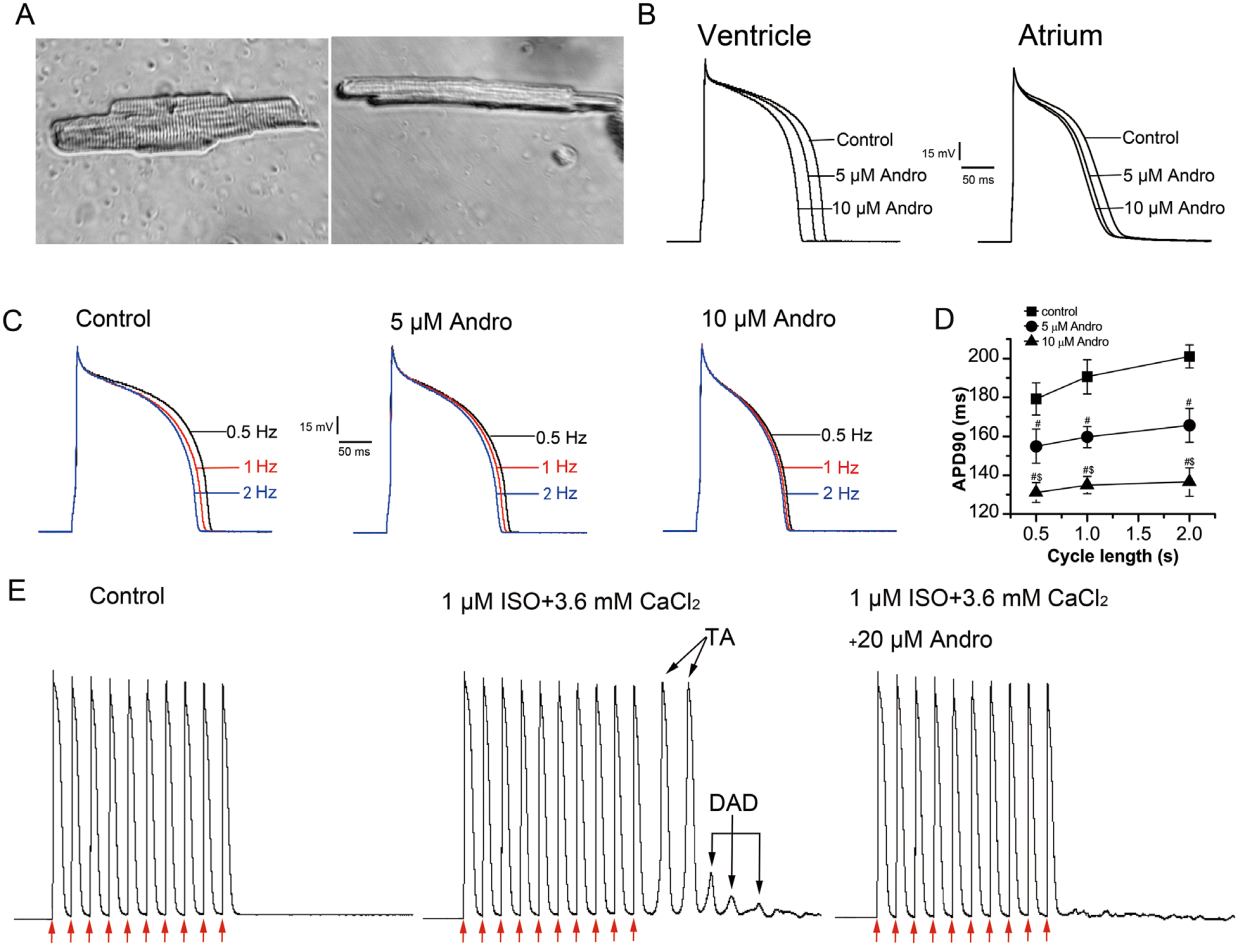
Effects of andrographolide (Andro) on APs, DADs, and DAD-induced TAs Typical photomicrographs of a single LVM (left) or LAM (right) cell under 40× light microscope **(A)**. Effects of andrographolide (5 or 10 μM) on APs recorded from rabbit LVMs (left) and LAMs (right) **(B)**. Effect of andrographolide (5 or 10 μM) on APs stimulated at 0.5, 1, or 2 Hz **(C)**. APs from 30 consecutive sweeps were averaged. The data of APD_90_ recorded at different stimulation frequencies are shown; andrographolide shortened APD_90_ and attenuated its RD in a concentration-dependent manner in LVMs **(D)**. Data are shown as means±SD (n=9, ^#^p<0.01 *vs* control, ^$^p<0.01 *vs* 5 μM andrographolide). Andrographolide abolished ISO (1 μM) and high calcium ([Ca^2+^]_o_=3.6 mM)-induced DADs and TAs in LVMs **(E)**. Arrows indicate depolarizing pulse.

In this study, left ventricular myocytes (LVMs) and left atrial myocytes (LAMs) were isolated at the same time and were tested alternately, so comparisons between LVM and LAM electrical activity parameters were reasonable. LVMs (Figure 1A, left) were larger and wider in size than LAMs (right), and had more intensive transverse striations.

APs were elicited by depolarizing pulses delivered in a width of 4 ms, 1.5-fold above the threshold at a rate of 1 Hz using the current-clamp technique. Figure 1B shows typical LVM (left) and LAM (right) morphology, and the effect of andrographolide on APs in these cells. Andrographolide shortened action potential duration at 50% repolarization (APD_50_) and 90% repolarization (APD_90_), and decreased maximum upstroke velocity (V_max_) in a concentration-dependent manner without changing resting membrane potential (RMP) or action potential amplitude (APA) in LVMs or LAMs. The effects of andrographolide on AP parameters were the same for LVMs and LAMs (Table 1).

**Table 1:** Effects of andrographolide on LVM and LAM AP parameters

Parameter	Andrographolide					
	Control		5 μM		10 μM	
	Ventricle	Atrium	Ventricle	Atrium	Ventricle	Atrium
RMP	-88 ± 2	-77 ± 4	-88 ± 2	-77 ± 5^+^	-88 ± 2	-77 ± 6^+^
APA	129 ± 4	129 ± 11	129 ± 5	128 ± 11^+^	128 ± 4	126 ± 11^+^
APD_50_	172 ± 7	91 ± 4	147 ± 8^##^	77 ± 6^##+^	131 ± 6^##$$^	66 ± 4^##$$+^
APD_90_	195 ± 8	137 ± 8	164 ± 7^##^	119 ± 6^#+^	142 ± 3^##$$^	111 ± 6^##$*^
V_max_	175 ± 7	249 ± 10	156 ± 8^# #^	226 ± 5^##+^	143 ± 7^##^	213 ± 7^##$+^

Data are presented as means±SD (ventricle, n=15; atrium, n=8).

^##^p<0.01 *vs* control ^#^p<0.05 *vs* control ^$$^p<0.01 *vs* 5 μM andrographolide ^$^p<0.05 *vs* 5 μM andrographolide ^+^p>0.05 *vs* ventricle ^*^p<0.05 *vs* ventricle.

RMP: resting membrane potential (mV); APA: action potential amplitude (mV); APD_50_: action potential duration at 50% repolarization (ms); APD_90_: action potential duration at 90% repolarization (ms); V_max_: maximum upstroke velocity (mV/ms).

### Effects of andrographolide on rate-dependent repolarization of APs in LVMs

APs were recorded using depolarizing pulses delivered in a width of 4 ms, 1.5-fold above the threshold at a rate of 1 Hz using the current-clamp technique. APs were recorded at stimulation frequencies of 0.5, 1, and 2 Hz with or without andrographolide after the AP stabilized at 1 Hz. Andrographolide shortened APD_90_ in a concentration-dependent manner and attenuated rate-dependence (RD) of APD (Figure 1C–1D).

### Effects of andrographolide on isoproterenol- and high calcium-induced delayed afterdepolarizations and triggered activities

APs were elicited by string stimulation containing 10 pulses at a basic cycle length (BCL) of 300 ms and string stimulation frequency of 0.125 Hz. APs were recorded after stabilization. Isoproterenol (ISO, 1 μM) was added to the external bath solution and the calcium concentration ([Ca^2+^]_o_) was increased to 3.6 mM at the same time. Under control circumstances, there were no delayed afterdepolarizations (DADs) or triggered activities (TAs). In contrast, ISO (1 μM) and high calcium ([Ca^2+^]_o_=3.6 mM) induced DADs and TAs in 7/7 LVMs following 10 consecutive pulses at a BCL of 300 ms (Figure 1E). The addition of 20 μM andrographolide abolished TAs and markedly reduced or abolished DADs.

### Effects of andrographolide on sodium current (I_Na_) in LVMs and LAMs

**Figure 2:**
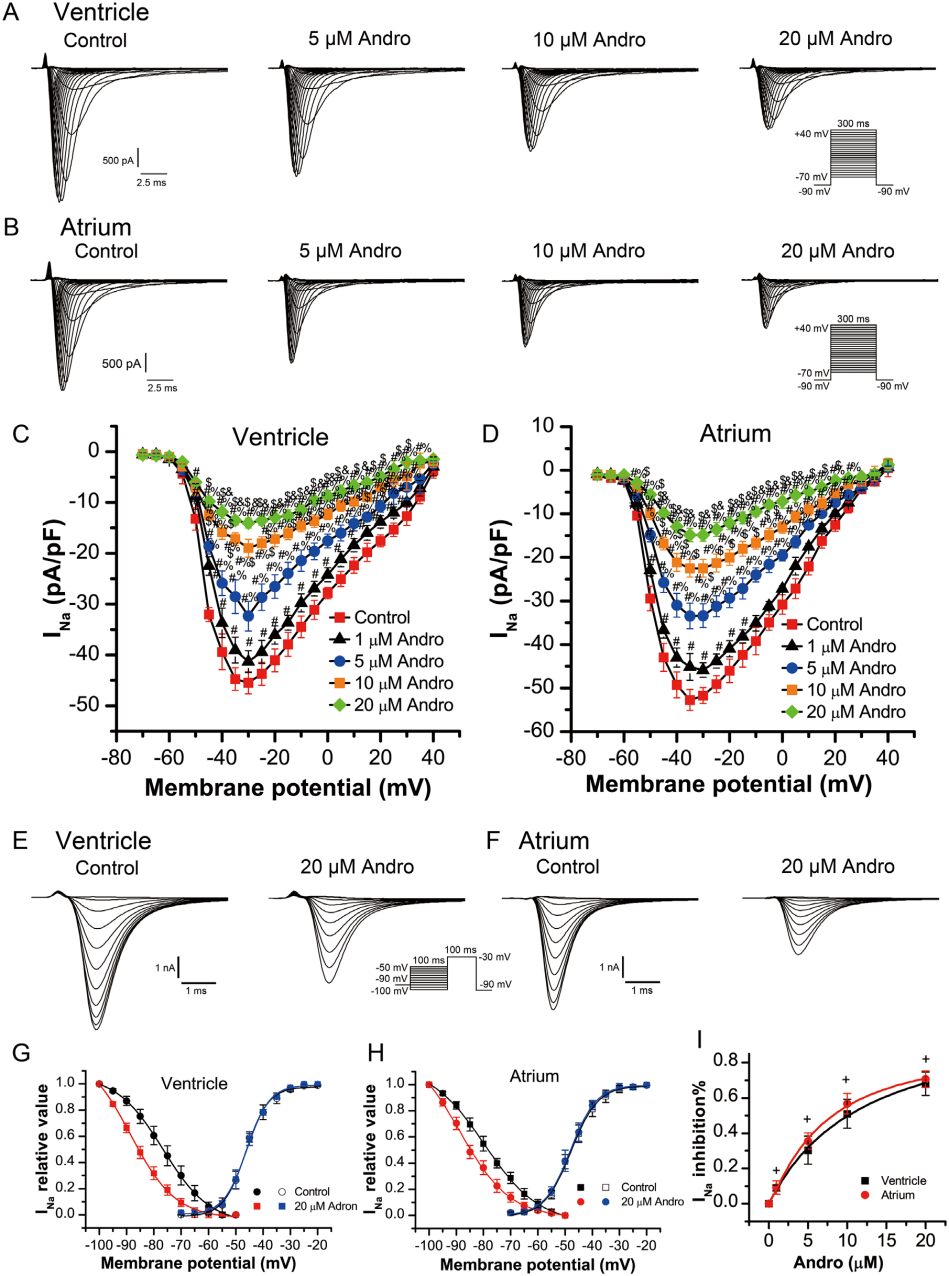
Andrographolide inhibited I_Na_ in a concentration dependent manner in LVMs and LAMs Representative whole-cell recordings of I_Na_ in LVMs **(A)** and LAMs **(B)** with or without 1, 5, 10, or 20 μM andrographolide. Current voltage relationships for I_Na_ in LVMs **(C)** and LAMs **(D)** Data are shown as means±SD (ventricle, n=10; atrium, n=8). ^#^p<0.01 *vs* control, ^%^p<0.01 *vs* 1 μM, ^$^p<0.01 *vs* 5 μM, ^&^p<0.01 *vs* 10 μM andrographolide. Representative I_Na_ recordings before **(E)** and after **(F)** andrographolide treatment using the inactivation protocol in the inset. Steady-state activation and inactivation curves for I_Na_ in LVMs **(G)** and LAMs **(H)** with or without 20 μM andrographolide. Lines represent data fit to a Boltzmann distribution function. Dose-reaction relationship between andrographolide and percent inhibition of I_Na_ (ventricle, n=10; atrium, n=10) **(I)**^+^p>0.05 *vs* ventricle.

I_Na_ was recorded using 300 ms depolarizing voltage steps between -70 mV and +40 mV in 5 mV increments from a holding potential of -90 mV at a rate of 0.5 Hz with the cells perfused with normal bath solution and recorded the current as control group. After currents stabilized, cells were perfused with solution containing andrographolide (1, 5, 10, or 20 μM). When sealing resistance was stable, currents were recorded under different concentrations and different voltages. Results in a single cell (Figure 2A–2B) and in a group cell (Figure 2C–2D) with or without andrographolide in LVMs and LAMs showed that andrographolide decreased I_Na_ in a concentration-dependent manner without shifting the voltage at which I_Na_ amplitude was maximal.

Steady-state inactivation curves for sodium currents in the absence or presence of 20 μM andrographolide were evaluated from a holding potential of -90 mV. A 100 ms conditioning pre-pulse was applied from -100 mV to +40 mV in 5 mV increments followed by 100 ms depolarizing test pulses to -30 mV at a rate of 0.5 Hz to evoke I_Na_. Figure 2E–2F show typical examples of one single cell from LVMs and LAMs, respectively. Figure 2G shows steady-state activation and inactivation curves fitted with a Boltzmann equation for I_Na_ under control conditions and after 20 μM andrographolide treatment in LVMs. For the steady-state activation of I_Na_, with and without 20 μM andrographolide, V_1/2_ values were -46.28±0.31mV and -45.97±0.30 mV (n=8, p>0.05), with k=4.13±0.28 mV and 4.21±0.27 mV (n=8, p>0.05), respectively. For the steady-state inactivation of I_Na_, with and without 20 μM andrographolide, V_1/2_ values were -76.63±0.38 mV and -88.52±0.84 mV (n=8, p<0.01), with k=8.60±0.45 mV and 8.25±0.53 mV (n=8, p>0.05), respectively.

Figure 2H shows the steady-state activation and inactivation curves fitted with a Boltzmann equation for I_Na_ with or without 20 μM andrographolide in LAMs. For the steady-state activation of I_Na_, with or without 20 μM andrographolide, V_1/2_ values were -47.81±0.33 mV and -48.03±0.21 mV (n=8, p>0.05), with k=4.93±0.31 mV and 4.83±0.19 mV (n=8, p>0.05), respectively. For the steady-state inactivation of I_Na_, with or without 20 μM andrographolide, V_1/2_ values were -79.58±0.39 mV and -88.64±0.04 mV (n=8, p<0.01), with k=9.97±0.45 mV and 9.17±0.65 mV (n=8, p>0.05), respectively. While andrographolide did not affect activation, but the voltage dependence of I_Na_ inactivation curve was shifted toward negative membrane potential, and andrographolide accelerated the inactivation process without changing its slope factor (Figure 2G–2H). Andrographolide inhibited I_Na_ in LVMs and LAMs with an IC_50_ of 10.41±3.18 μM and 8.28±1.26 μM, respectively (Figure 2I). The effects of andrographolide on I_Na_ were similar between LVMs and LAMs (ventricle, n=10; atrium, n=10; p>0.05 *vs* ventricle).

### Effects of andrographolide on L-type calcium current (I_CaL_) in LVMs and LAMs

**Figure 3:**
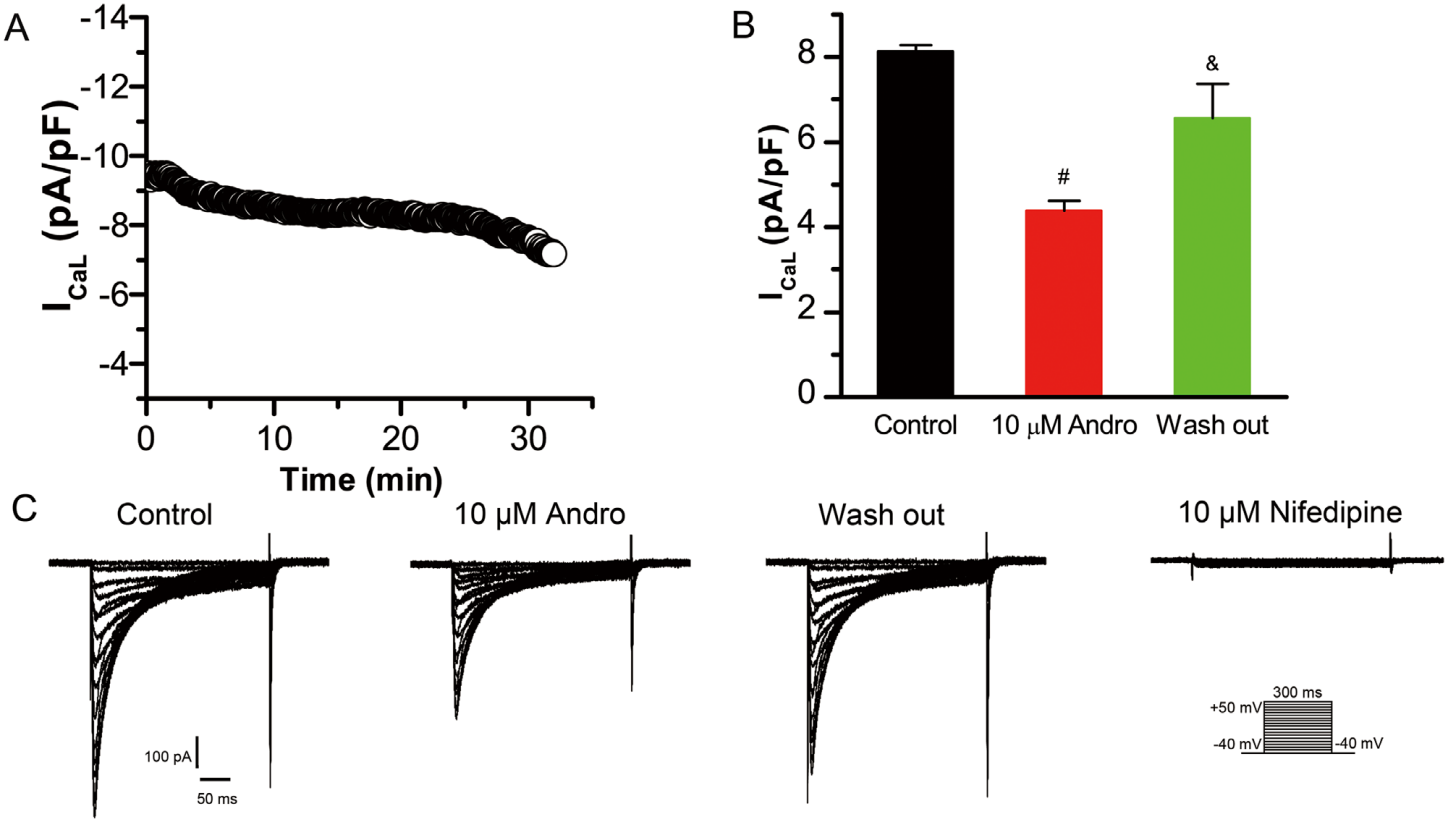
Andrographolide inhibition of I_CaL_ is reversible I_CaL_ time course after membrane rupture in LVMs **(A)**. Histograms of I_CaL_ current densities for control, 10 μM andrographolide, and washout (n=8) **(B)**. ^#^p<0.01 *vs* control, ^&^p<0.01 *vs* 10 μM andrographolide). Data are shown as means±SD. Effects of andrographolide on I_CaL_ are reversible, and nifedipine (10 μM) blocked I_CaL_ completely in LVMs **(C)**.

I_CaL_ was recorded using a 300 ms depolarizing pulse from -40 mV to +50 mV in 5 mV increments at a rate of 0.5 Hz with a holding potential of -40 mV. One of the difficulties of studying I_CaL_ is the run-down phenomenon in which the membrane ruptures. Figure 3A shows I_CaL_ current density changes over time after membrane rupture without addition of any drug in LVMs. I_CaL_ tends to stabilize at 7–25 min after membrane rupture. To show that andrographolide inhibition of I_CaL_ was independent of the run-down phenomenon, the drug was added after I_CaL_ peak amplitude stabilized (about 7 min after membrane rupture). The experiment was accomplished within 25 min after membrane rupture to avoid interference by the run-down phenomena.

Figure 3C shows typical I_CaL_ current traces from a single LVM cell under control conditions or with 10 μM andrographolide, washout, and 10 μM nifedipine. 10 μM andrographolide inhibited I_CaL_, and I_CaL_ amplitude recovered progressively after withdrawal of andrographolide (washout). Then, to identify I_CaL_, current was recorded after addition of 10 μM nifedipine. I_CaL_ was almost completely blocked by 10 μM nifedipine, indicating that the current recorded with the above pulse stimulation was I_CaL_. These results demonstrated that andrographolide inhibition of I_CaL_ is significant and reversible (Figure 3B–3C).

**Figure 4:**
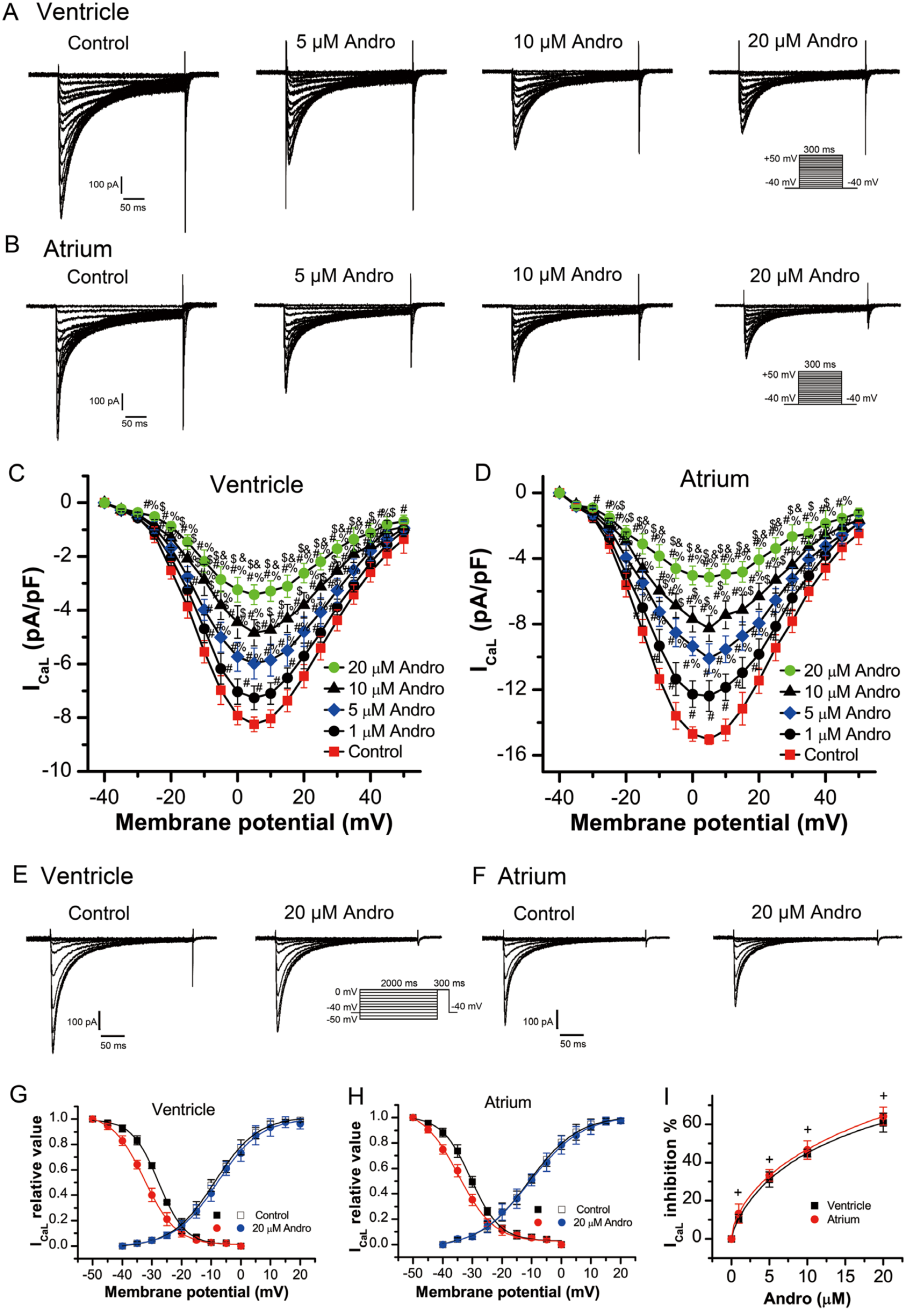
Andrographolide inhibited I_CaL_ in a concentration-dependent manner in LVMs and LAMs Representative whole-cell recordings of I_CaL_ in LVMs **(A)** and LAMs **(B)** with or without 1, 5, 10, or 20 μM andrographolide. Current voltage relationships for I_CaL_ in LVMs **(C)** and LAMs **(D)** Data are shown as means±SD (ventricle, n=14; atrium, n=15). ^#^p<0.01 *vs* control, ^%^ p<0.01 *vs* 1 μM, ^$^ p<0.01 *vs* 5 μM, ^&^p<0.01 *vs* 10 μM andrographolide. Representative I_CaL_ recordings before **(E)** and after **(F)** andrographolide treatment using the inactivation protocol in the inset.Steady-state activation and inactivation curves for I_CaL_ in LVMs **(G)** and LAMs **(H)** with or without 20 μM andrographolide. Lines represent the data fit to a Boltzmann distribution function. Dose-reaction relationship between andrographolide and percent inhibition of I_CaL_ (ventricle, n=8; atrium, n=10) **(I)** ^+^p>0.05 *vs* ventricle.

Figure 4A–4B shows typical I_CaL_ current traces from single LVM or LAM cells before (control) and after andrographolide treatment. I_CaL_ current amplitude tended to stabilize within 1 min after andrographolide addition. Results in a typical single LVM or LAM cell (Figure 4A–4B) and in a group cell (Figure 4C–4D) with or without andrographolide showed that andrographolide inhibited I_CaL_ in a concentration-dependent manner. Figure 4C–4D shows the I_CaL_ I–V relationship after andrographolide application, which indicated that andrographolide inhibited I_CaL_ without shifting the voltage at which I_CaL_ amplitude was maximal.

The steady-state I_CaL_ inactivation curves with or without 20 μM andrographolide were evaluated from a holding potential of -40 mV. A 2000 ms conditioning pre-pulse was applied from -50 mV to 0 mV in 5 mV increments followed by 300 ms depolarizing test pulses to 0 mV at a rate of 0.5 Hz to evoke I_CaL_. Figure 4E–4F show typical example for one single cell of LVMs and LAMs, respectively. Figure 4G shows the steady-state activation and inactivation curves fitted with a Boltzmann equation for I_CaL_ under control conditions and after 20 μM andrographolide application in LVMs. For the steady-state activation of I_CaL_, with or without 20 μM andrographolide, V_1/2_ values were -9.26±0.44 mV and -8.53±0.53 mV (n=14, p>0.05), with k=6.69±0.41 mV and 6.51±0.49 mV (n=14, p>0.05), respectively. For the steady-state inactivation of I_CaL_, with or without 20 μM andrographolide, V_1/2_ values were -28.44±0.23 mV and -32.87±0.35 mV (n=7, p<0.01), with k=4.55±0.21 mV and 6.09±0.31 mV (n=7, p<0.01), respectively. Figure 4H shows the steady-state activation and inactivation curves fitted with a Boltzmann equation for I_CaL_ with or without 20 μM andrographolide in LAMs. For the steady-state activation of I_CaL_, with or without 20 μM andrographolide, V_1/2_ values were -10.99±0.31 mV and -10.66±0.33 mV (n=13, p>0.05), with k=8.32±0.33 mV and 8.89±0.37 mV (n=13, p>0.05), respectively. For the steady-state inactivation of I_CaL_, with or without 20 μM andrographolide, V_1/2_ values were -30.54±0.29 mV and -34.68±0.54 mV (n=8, p<0.01), with k=4.93±0.27 mV and 6.02±0.45 mV (n=8, p<0.01), respectively. These results suggest that andrographolide did not affect I_CaL_ activation, but shifted the voltage dependence of I_CaL_ inactivation curve toward negative membrane potential. Andrographolide also accelerated the inactivation process and increased its slope factor (Figure 4G–4H). Andrographolide inhibited I_CaL_ in LVMs and LAMs with an IC_50_ of 11.91±1.55 μM and 10.08±1.15 μM, respectively (Figure 4I). Andrographolide had similar effects on I_CaL_ between LVMs and LAMs (ventricle, n=8; atrium, n=10. p>0.05 *vs* ventricle).

### Effect of andrographolide on inward rectifying potassium current (I_k1_) in LVMs

**Figure 5:**
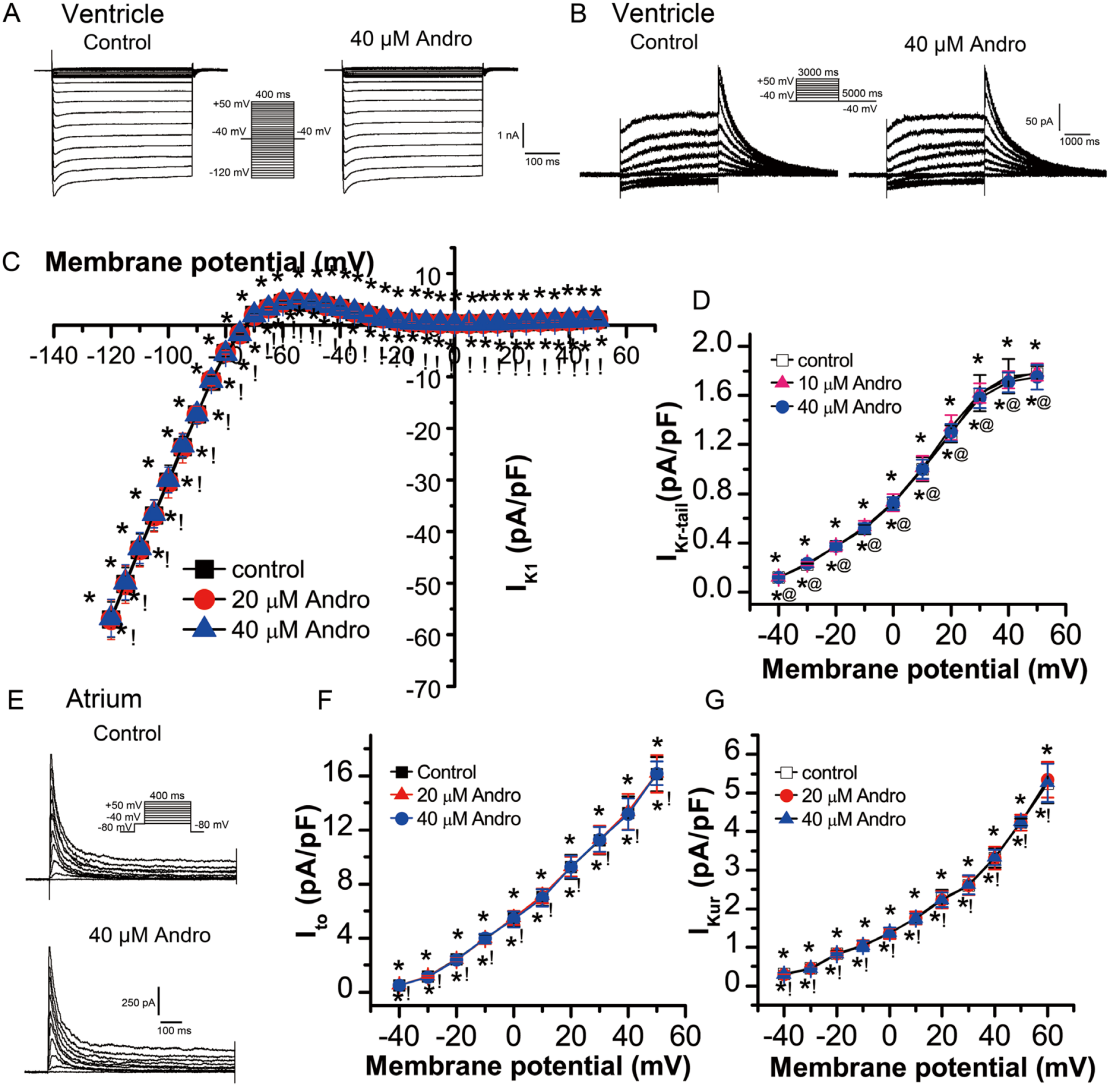
Effects of andrographolide on I_K1_, I_Kr_, I_to_, and I_Kur_ I_K1_ **(A)** and its I-V relationship **(C)** in LVMs beforeand after andrographolide treatment. Data are shown as means±SD (n=14). Typical I_Kr_ current traces from a single LVM cell **(B)** and its I-V relationship **(D)** before and after andrographolide treatment (n=15). Representative I_to_ current recording **(E)** and its I-V relationship **(F)** in LAMs before and after andrographolide treatment (n=15). I_Kur_ I-V relationship in LAMs before and after application of andrographolide (n=12) **(G)**. *p>0.05 *vs* control, ^@^p>0.05 *vs* 10 μM andrographolide, ^!^p>0.05 *vs* 20 μM andrographolide.

I_K1_ was elicited by 400 ms depolarization voltage from -120 mV to +50 mV in 5 mV increments at a rate of 1 Hz with a holding potential of -40 mV. Figure 5A shows typical I_K1_ current traces from a single cell before and after 40 μM andrographolide treatment. Neither 20 μM nor 40 μM andrographolide affected I_K1_ (Figure 5C).

### Effect of andrographolide on rapid delayed rectifier potassium current (I_Kr_) in LVMs

I_Kr_ was elicited by 3000 ms depolarization voltage from -40 mV to +50 mV in 10 mV increments followed by a 5000 ms repolarization pulse to -40 mV at a rate of 0.1 Hz with a holding potential of -40 mV. Figure 5B shows typical single cell I_Kr_ current traces before and after 40 μM andrographolide application. Neither 10 μM nor 40 μM andrographolide affected I_Kr_ (Figure 5D).

### Effect of andrographolide on transient outward potassium current (I_to_) in LAMs

I_to_ was recorded using a 400 ms depolarization voltage from -40 mV to +50 mV in 10 mV increments at a rate of 0.1 Hz with a holding potential of -80 mV. I_Na_ was eliminated by 100 ms, with -40 mV depolarizing pre-pluses. Figure 5E shows typical I_to_ current traces from a single cell before and after 40 μM andrographolide application. Neither 20 μM nor 40 μM andrographolide affected I_to_ (Figure 5F).

### Effect of andrographolide on ultra-rapid delayed rectifier potassium current (I_Kur_) in LAMs

I_Kur_ was recorded with an 80 ms pre-pulse to +30 mV to inactivate I_to_, then repolarized to -50 mV, lasting 50 ms, followed by a 150 ms test pulse to +60 mV from -40 mV in 10 mV increments, at a rate of 0.5 Hz with a holding potential of -50 mV. Neither 20 μM nor 40 μM andrographolide affected I_Kur_ (Figure 5G).

### Effect of andrographolide on aconitine-induced ventricular arrhythmias in rabbits

**Figure 6:**
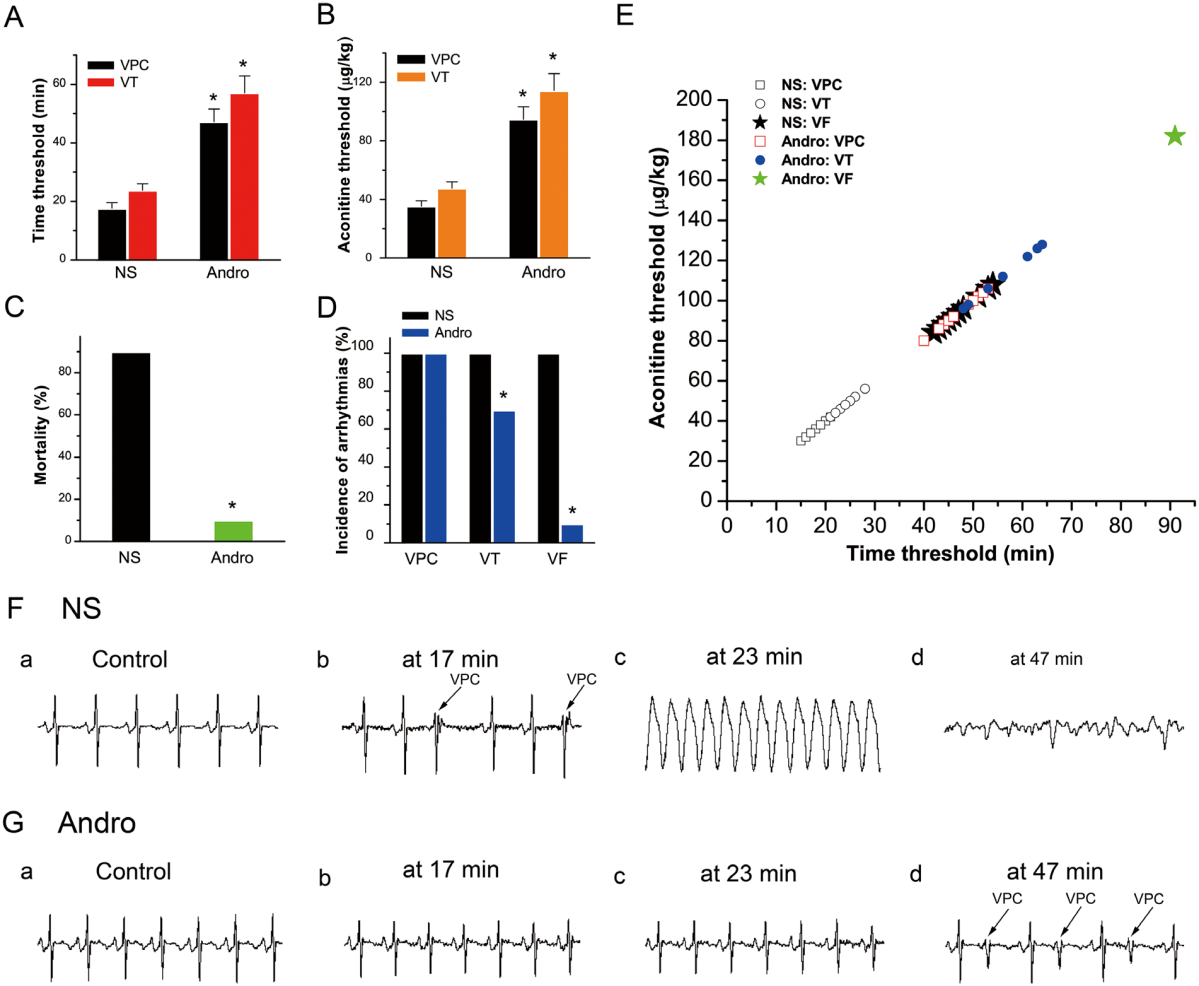
Effects of andrographolide on aconitine-induced arrhythmias Histogram of the aconitine-induced arrhythmia time threshold (NS: n=10; Andro: VPC, n=10; VT, n=7) (A). Histogram of the aconitine threshold (NS: n=10; Andro: VPC, n=10; VT, n=7) (B). VPC and VT appeared in all 10 NS group rabbits, and in 10 and 7 Andro group rabbits, respectively. Rabbit mortality before and after andrographolide treatment (NS: n=10, Andro: n=10) **(C)**. Incidence of aconitine-induced multiple ventricular arrhythmias in the two groups (D). VPC was triggered successfully in all animals, but VT and VF incidences were reduced in the Andro group. Time-dosage threshold curve of various types of ventricular arrhythmias **(E)**. VPC, VT and VF were observed in all 10 NS group rabbits, and in 10, 7, and 1 of 10 Andro group rabbits, respectively. Typical ECG tracings showing various types of ventricular arrhythmias before **(Fa)** and after aconitine treatment (2 μg/kg/min) **(Fb–d)** VPC, VT, and VF began to appear at 17, 23, and 47 min (F). Typical ECG tracings before (Ga) and after andrographolide (10 mg/kg) and aconitine (2 μg/kg/min) treatment **(Gb–d)**. The times of the four ECG tracings in **(G)** were consistent with those in **(F)**. After andrographolide treatment, VPC appeared at 47 min, and arrhythmia did not appear at either 17 or 23 min. *p<0.01 *vs* NS. NS: normal saline; VPC: ventricular premature contraction; VT: ventricular tachycardia; VF: ventricular fibrillation.

Ventricular premature contraction (VPC), ventricular tachycardia (VT), and ventricular fibrillation (VF) appeared in all 10 NS group rabbits, and in 10, 7, and 1 of 10 Andro group rabbits, respectively. Andrographolide treatment prior to aconitine increased the time threshold (Figure 6A) and aconitine cumulative dosage (Figure 6B) required to induce VPC, VT, and VF compared with the NS group. Andrographolide treatment also reduced rabbit mortality (Figure 6C) and aconitine-induced ventricular arrhythmia incidence (Figure 6D–6E).

## DISCUSSION

This study found that andrographolide inhibited I_Na_ and I_CaL_ in LVMs and LAMs in a concentration-dependent manner. Andrographolide (5 and 10 μM) decreased APD_50_, APD_90_, and V_max_ without affecting RMP and APA in these cells. Treatment also shortened APD and attenuated RD of APD in LVMs in a concentration-dependent manner. We found that 20 μM andrographolide attenuated TAs and DADs induced by ISO (1 μM) and high calcium (3.6 mM) in LVMs. Finally, andrographolide had no influence on I_K1_ and I_Kr_ in LVMs, or I_to_ and I_Kur_ in LAMs. Our results suggest that andrographolide had cardioprotective effects in rabbits with aconitine-induced arrhythmias.

Andrographolide shifted the I_Na_ inactivation curve toward negative membrane potential and accelerated the inactivation process without changing its slope factor. Decreased I_Na_ amplitude and accelerated I_Na_ inactivation indicated that the number of Na^+^ flowing into the cell was reduced. Since the transient sodium current initiates the action potential [12], increasing the quantity of current passed through the membrane would enhance cell excitability. Thus, andrographolide decreased cell excitability, which suggests its potential use in treatment of arrhythmias caused by enhanced excitability. In cardiomyocytes, intracellular Na^+^ concentration ([Na^+^]_i_) is an important Ca^2+^ homeostasis and cell contractility modulator [13]. [Na^+^]_i_ is increased under certain pathological conditions, such as myocardial ischemia [14]. In this study, andrographolide inhibited I_Na_. Therefore, andrographolide reversed increased [Na^+^]_i_ levels under pathological conditions by inhibiting I_Na_, which could help alleviate the Na^+^-dependent calcium overload in cardiomyocytes under some pathological conditions [15]. These results indicate that andrographolide might serve as a class I antiarrhythmic, cardioprotective therapeutic.

Normal I_CaL_ regulation is key to intracellular Ca^2+^ ([Ca^2+^]_i_) homeostasis. The inorganic calcium ion (Ca^2+^), an important messenger in living cells, is responsible for signal transduction and regulation of vital functions [16]. Disruption of [Ca^2+^]_i_ homeostasis (calcium overload) is closely related to multiple pathological conditions, including arrhythmia [17], myocardial ischemia/reperfusion [18], heart failure [19], digitalis intoxication [20], and hypercalcinemia [21]. Andrographolide inhibited I_CaL_ concentration-dependently, and shifted the I_CaL_ inactivation curve toward negative membrane potential by amplifying its V_1/2_ and k values. When k was increased, the inactivation process accelerated. I_CaL_ inactivation acceleration and decreased I_CaL_ amplitude indicated that the number of Ca^2+^ flowing into cell was reduced. Since andrographolide decreased [Ca^2+^]_i_, it might alleviate Ca^2+^ overloading in cardiomyocytes. Andrographolide could inhibit increased [Ca^2+^]_i_ and restore intracellular Ca^2+^ homeostasis, suggesting its potential use as a class IV anti-arrhythmic drug.

Andrographolide did not influence rabbit cardiomyocyte RMP, which was consistent with our finding that it had no effect on LVM I_K1_. Andrographolide (5 and 10 μM) had no effect on APA, but reduced V_max_. A V_max_ reduction can decrease cardiomyocyte excitability and slow down the excitement conduction velocity, which is helpful for inhibiting reentrant tachycardia. Since andrographolide had no influence on I_K1_, I_Kr_, I_to_, or I_Kur_, andrographolide mainly shortens APD_50_ and APD_90_ by inhibiting I_CaL_. In this study, andrographolide not only shortened AP duration, but also decreased the RD of APD concentration-dependently. When RD decreased, the transmural dispersion of repolarization also diminished. Therefore, andrographolide may reduce the transmural repolarization heterogeneity of APD. Enlargement of APD transmural repolarization heterogeneity leads to the occurrence of arrhythmias [22]. Andrographolide reduced RD, indicating that it may reduce the transmural repolarization heterogeneity of APD, thus also inhibiting reentrant arrhythmias.

ISO and high calcium causes Ca^2+^-overload by increasing [Ca^2+^]_i_, thereby resulting in DADs and TAs [23–25]. DAD-mediated TA can increase abnormal autorhythmicity, causing the majority of sudden cardiac deaths during nonischemic heart failure [24, 25]. In this study, 20 μM andrographolide inhibited DADs and TAs evoked by ISO and high calcium. The potential mechanism may be related to andrographolide inhibition of I_CaL_ and I_Na_ to alleviate intracellular Ca^2+^- and Na^+^-overloading.

This study also found that andrographolide has no effect on I_K1_, I_Kr_, I_to_, and I_Kur_. Enhanced reverse rate-dependence (RRD) of APD caused by drugs that inhibit I_Kr_ is an undesirable response, as it reduces drug efficacy during tachycardia and may promote proarrhythmic activity after tachycardia termination [26, 27]. Andrographolide as a potential antiarrhythmia therapeutic does not induce such side effects.

Finally, *in vivo* results indicated that andrographolide had cardioprotective effects in rabbits with aconitine-induced arrhythmias. Aconitine binds to voltage-dependent cardiac Na^+^ channels [28, 29] and prolongs their open state, favoring entry of a large quantity of Na^+^ into cytosol. This may be accompanied by Ca^2+^ overload via an electrogenic Na^+^-Ca^2+^ exchange (NCX) system, and eventually induces triggered activity [30, 31]. Furthermore, aconitinine can increase I_CaL_ by accelerating the activation process and delaying inactivation process [32, 33]. We found that andrographolide increased the time threshold and cumulative aconitine dosage required to induce VPC, VT, and VF. It also reduced incidences of aconitine-induced VT and VF, as well as mortality in rabbits. These results indicated that andrographolide had the protective effects on aconitine-induced arrhythmias. And this antiarrhythmic effect of andrographolide was related to its inhibition of I_Na_ and I_CaL_.

To the best of our knowledge, this is the first demonstration about the effects of andrographolide on cardiac myocyte electrophysiologyical properties, and its possible antiarrhythmic mechanisms is related to its inhibitory effects on I_Na_ and I_CaL_. Our results of this study indicate that andrographolide may be a potential class I and IV antiarrhythmia agent.

## MATERIALS AND METHODS

### Ethics statement

Use of rabbits in this investigation conformed to the Guide for the Care and Use of Laboratory Animals published by the National Institutes of Health (NIH publication no. 85-23, revised 1996) and the Guide for the Care and Use of Laboratory Animals (Hubei Province, China), and was approved by the Institutional Animal Care and Use Committee of Wuhan University of Science and Technology.

### Cardiomyocyte isolation

Single cardiac ventricular and atrial myocytes were enzymatically isolated from adult New Zealand white rabbits (1.7–2 kg weight, 24–32 weeks of age, of either sex), as described preciously [34]. Rabbits were provided by the Experimental Animal Center of Wuhan University of Science and Technology. Rabbits were heparinized (2000 U) and anesthetized with ketamine (30 mg/kg, intravenously) and xylazine (7.5 mg/kg, intramuscularly). After the corneal reflex disappeared, rabbits were fixed on their backs to the test stands. Hearts were quickly removed after thoracotomy and put in a petri dish filled with 37°C, oxygen (95% O_2_ and 5% CO_2_) saturated, Ca^2+^- free Tyrode’s solution containing (mM): NaCl 135, NaH_2_PO_4_ 0.33, KCl 5.4, MgCl_2_ 1.0, HEPES 10, glucose 10 (pH 7.4, adjusted with NaOH) to stop the heartbeat. Fat and connective tissues were removed from the heart. To eliminate residual blood in the heart cavity, the aorta was cannulated and perfused retrogradely using a modified Langendorff apparatus (perfusion pressure 70 cm H_2_O_2_), then perfused for 5 more min with oxygen saturated, Ca^2+^-free Tyrode’s solution (at 37°C). Hearts were then digested for 30 min with the same solution containing enzyme (collagenase type I, 1 g/L) and bovine serum albumin (BSA, 1 g/L) at 37°C. The enzyme-containing solution was pumped at a constant rate using a peristaltic pump, and gas (95% O_2_ and 5% CO_2_) was allowed to pass through this solution. The perfusate was then switched to preheated and oxygen saturated Kraftbrühe (KB) solution (mM): KOH 70, KCl 40, KH_2_PO_4_ 20, glutamic acid 50, taurine 20, EGTA 0.5, glucose 10, HEPES 10, MgSO_4_ 3 (pH 7.4, adjusted with KOH) for 5 min to replace the Ca^2+^-free Tyrode’s solution. All solutions were bubbled with 95% O_2_ and 5% CO_2_ and maintained at 37°C. The left ventricle and left atrium were then cut into small chucks separately and gently agitated in KB solution (BSA, 1 g/L). Cells were filtered using nylon mesh and stored in KB solution (BSA, 1 g/L) until use.

### Aconitine-induced arrhythmias in rabbits

Adult New Zealand white rabbits were randomly divided into two groups (n=10, each): i) Normal saline (NS) group; ii) andrographolide (Andro) group. Rabbits were then anesthetized and fixed on their backs to test stands. Polyethylene tubing was inserted into the ear marginal vein to administer aconitine, NS, and andrographolide. Standard limb lead II electrocardiogram (ECG) was measured using the BL-420F data acquisition and analysis system (Chengdu TaiMeng, Sichuan, China) following subcutaneous penetration of electrodes into four limbs. After 10 min of stabilization, NS (10 ml) was injected into NS group rabbits in 5 min through the ear marginal vein. Five min later, aconitine (2 μg/kg/min, flow rate: 80 μl/min) was injected using an infusion pump to induce arrhythmias. Andro group rabbits were treated with 10 ml andrographolide (10 mg/kg) in 5 min prior to aconitine (2 μg/kg/min, flow rate: 80 μl/min). ECG recordings were conducted in both groups for 120 min following aconitine administration. The cumulative dosage of aconitine required to induce VPC, VT and VF was calculated. VPC, VT, and VF time thresholds were recorded.

### Drugs and solutions

Collagenase type I and CsCl were purchased from Gibco (Invitrogen Co., Paisley, UK). Bovine serum albumin (BSA) and HEPES were obtained from Roche (Basel, Switzerland). L-glutamic acid and taurine were purchased from Wuhan Zhongnan Chemical Reagent Co. (Wuhan, China). E-4031 and chromanol 293B were obtained from Tocris Bioscience (Minneapolis, MN, USA). All other drugs and reagents were purchased from Sigma Aldrich (Saint Louis, MO, USA). Andrographolide was dissolved in methyl alcohol, chromanol 293B in DMSO, and nifedipine in ethyl alcohol. All drugs were dissolved and diluted into the external recording solution immediately before use. Final methyl alcohol, DMSO, and ethyl alcohol concentrations in the test solutions were ≤0.1% to assure no effects on recorded currents. Stock solution of other drugs were prepared in dH_2_O. Drug concentrations refer to that in the external recording solution.

The AP recording bath solution contained (mM): NaCl 140, KCl 4.0, CaCl_2_ 1.8, MgCl_2_ 1.0, HEPES 5.0, Glucose 10.0 (pH 7.4, adjusted with NaOH) and the pipette solution contained (mM): K-aspartate 110, KCl 30, HEPES 10, EGTA 0.1, MgATP 5, Creatine phosphate 5, CAMP 0.05 (pH 7.2, adjusted with KOH).

The I_Na_ recording bath solution contained (mM): NaCl 30, CaCl_2_ 1.0, CsCl 105, MgCl_2_ 1.0, HEPES 5.0, Glucose 5 (pH 7.4, adjusted with CsOH) and the intracellular pipette solution contained (mM): CsCl 120, Na_2_ATP 5, MgCl_2_ 5, CaCl_2_ 1.0, TEA-Cl 10, EGTA 10, HEPES 10 (pH 7.3, adjusted with CsOH). Nifedipine (0.01 mM) was added to the bath solution to block L-type Ca^2+^ channels.

The I_CaL_ recording bath solution contained (mM): NaCl 135, CsCl 5.4, CaCl_2_ 1.8, MgCl_2_ 1.0, BaCl_2_ 0.3, NaH_2_PO_4_ 0.33, Glucose 10, EGTA 10 (pH 7.4, adjusted with NaOH), and the pipette solution contained (mM): CsCl 120, Na_2_ATP 5, MgCl_2_ 5, CaCl_2_ 1.0, TEA-Cl 10, EGTA 10, HEPES 10 (pH 7.3, adjusted with CsOH).

Extracellular solution for I_K1_ recordings contained (mM): NaCl 137, KCl 5.4, CaCl_2_ 1.8, MgCl_2_ 1.0, NaH_2_PO_4_ 0.33, HEPES 10, Glucose 10, CdCl_2_ 0.3 (to block L-type Ca^2+^ channels) (pH 7.4, adjusted with NaOH). Intracellular solution for I_K1_ recordings contained (mM): KCl 140, MgCl_2_ 1.0, K_2_ATP 5, EGTA 10, HEPES 5.0 (pH 7.3, adjusted with KOH).

The I_to_ and I_Kur_ recording bath solution contained (mM): NaCl 140, KCl 5.4, MgCl_2_ 1.0, CaCl_2_ 1.8, NaH_2_PO_4_ 0.33, HEPES 5, Glucose 10 (pH 7.4, adjusted with NaOH) and the intracellular pipette solution contained (mM): KCl 20, K-aspartate 110, MgCl_2_ 1.0, HEPES 10, EGTA 5, GTP 0.1, Na_2_-phosphocreatine 5, Mg_2_ATP 5 (pH 7.2, adjusted with KOH). For recording I_to_ or I_Kur_, 200 μM BaCl_2_ and 200 μM CdCl_2_ were added to the bath solution to block I_K1_ and I_CaL_, respectively. Atropine (1.0 μM) was used to block I_K,Ach_. To inhibit sodium current, an inactivating pre-pulse (-40 mV, lasting 100 ms) was used when recording I_to_. While recording I_Kur_, sodium current was inhibited by a holding potential of 50 mV and equimolar N-methyl-D-glucamine replacement of Na^+^ in the bath solution.

For recording I_Kr_, the external solution contained (mM): NaCl 135, KCl 5.4, CaCl_2_ 1.0, CdCl_2_ 0.2, NaH_2_PO_4_ 0.33, MgCl_2_ 1.0, HEPES 5, Glucose 5 (pH 7.4, adjusted with NaOH). The intracellular pipette solution contained (mM): KCl 140, MgCl_2_ 1.0, HEPES 5, EGTA 10, Na_2_ATP 2 (pH 7.25, adjusted with KOH).

### Current recordings

Transmembrane action potentials and ion currents were recorded from isolated myocytes using a patch clamp amplifier (EPC-10, Heka Electronic, Lambrecht, Pfalz, Germany) with a current or voltage clamp technique, respectively. All experiments were performed at 37±0.5°C and bath solutions were bubbled with 95% O_2_ and 5% CO_2_. Before patch clamp recording, myocytes were transferred into a recording chamber mounted on an inverted microscope stage (Nikon TE2000-S) and allowed to adhere to the glass bottom of the chamber for 5 min. A motorized micromanipulator (MP285, Sutter, USA) propelled the recording electrode to the chosen cells. Membranes were then sealed and ruptured with negative pressure. Sealing resistance was maintained >1GΩ. A 80% compensation of series resistance was achieved without ringing. Capacitance and series resistances were adjusted to minimize the contribution of the capacitive transients. Patch electrodes were pulled with a two-stage patch pipette puller (PP-830, Narishige Group, Tokyo, Japan) and then fire-polished. Patch electrodes had a resistance of 1.8–2.5 MΩ when filled with pipette solution. Current signals were conducted by the Ag/AgCl electrode and amplified by an EPC-10 amplifier, then filtered at 1.5 kHz, digitized at 10 kHz, and stored on a computer hard disk for analysis.

### Data analysis

Applicate FitMaster (v2x32; HEKA) was used for data analysis and SPSS 18.0 for statistical analysis. Figures were plotted using Origin software (V7.0, OriginLab Co., MA, USA). Summary data were presented as means±SD. Current density was obtained by dividing current amplitude by cell capacitance. Data from steady-state I_Na_ activation and inactivation were fitted to the Boltzmann equation: Y=1/[1+exp(Vm -V_1/2_)/k], where Vm is the membrane potential, V_1/2_ the half-activation or half-inactivation potential, and k the slope factor. For steady-state activation curves, Y represents relative conductance. Chord conductance was calculated using the ratio of the current to electromotive force for potential in individual current-voltage relationships. Conductances were normalized to their individual maximal conductance. For steady-state inactivation curves, Y represents relative current (I_Na_/I_Na_max; I_CaL_/I_CaL_max). Differences between groups were determined using one-way analysis of variance (ANOVA) followed by the Tukey multiple comparison test or Student’s paired t-test. P<0.05 was considered statistically significant.

## References

[1] Ren J, Liu Z, Wang Q, Giles J, Greenberg J, Sheibani N, Kent KC, Liu B (2016). Andrographolide ameliorates abdominal aortic aneurysm progression by inhibiting inflammatory cell infiltration through downregulation of cytokine and integrin expression. J Pharmacol Exp Ther.

[2] Kishore V, Yarla NS, Bishayee A, Putta S, Malla R, Neelapu NR, Challa S, Das S, Shiralgi Y, Hegde G, Dhananjaya BL (2017). Multi-targeting andrographolide and its natural analogs as potential therapeutic agents. Curr Top Med Chem.

[3] Wong SY, Tan MG, Wong PT, Herr DR, Lai MK (2016). Andrographolide induces Nrf2 and heme oxygenase 1 in astrocytes by activating p38 MAPK and ERK. J Neuroinflammation.

[4] Xu F, Wu H, Zhang K, Lv P, Zheng L, Zhao J (2016). Proneurogenic effects of andrographolide on RSC96 Schwann cells *in vitro*. Mol Med Rep.

[5] Awang K, Abdullah NH, Hadi AH, Fong YS (2012). Cardiovascular activity of labdane diterpenes from Andrographis paniculata in isolated rat hearts. J Biomed Biotechnol.

[6] Woo AY, Waye MM, Tsui SK, Yeung ST, Cheng CH (2008). Andrographolide up-regulates cellular-reduced glutathione level and protects cardiomyocytes against hypoxia/reoxygenation injury. J Pharmacol Exp Ther.

[7] Trivedi NP, Rawal UM (2001). Hepatoprotective and antioxidant property of Andrographis paniculata (Nees) in BHC induced liver damage in mice. Indian J Exp Biol.

[8] Zhang J, Zhu D, Wang Y, Ju Y (2015). Andrographolide attenuates LPS-induced cardiac malfunctions through inhibition of IkappaB phosphorylation and apoptosis in mice. Cell Physiol Biochem.

[9] Lien LM, Su CC, Hsu WH, Lu WJ, Chung CL, Yen TL, Chiu HC, Sheu JR, Lin KH (2013). Mechanisms of andrographolide-induced platelet apoptosis in human platelets: regulatory roles of the extrinsic apoptotic pathway. Phytother Res.

[10] Lu WJ, Lin KH, Hsu MJ, Chou DS, Hsiao G, Sheu JR (2012). Suppression of NF-kappaB signaling by andrographolide with a novel mechanism in human platelets: regulatory roles of the p38 MAPK-hydroxyl radical-ERK2 cascade. Biochem Pharmacol.

[11] Wang YJ, Wang JT, Fan QX, Geng JG (2007). Andrographolide inhibits NF-kappaBeta activation and attenuates neointimal hyperplasia in arterial restenosis. Cell Res.

[12] Kuksis M, Ferguson AV (2015). Actions of a hydrogen sulfide donor (NaHS) on transient sodium, persistent sodium, and voltage-gated calcium currents in neurons of the subfornical organ. J Neurophysiol.

[13] Bay J, Kohlhaas M, Maack C (2013). Intracellular Na(+) and cardiac metabolism. J Mol Cell Cardiol.

[14] Williams IA, Xiao XH, Ju YK, Allen DG (2007). The rise of [Na(+)] (i) during ischemia and reperfusion in the rat heart-underlying mechanisms. Pflugers Arch.

[15] Zhang S, Ma JH, Zhang PH, Luo AT, Ren ZQ, Kong LH (2012). Sophocarpine attenuates the Na(+)-dependent Ca2(+) overload induced by Anemonia sulcata toxin-increased late sodium current in rabbit ventricular myocytes. J Cardiovasc Pharmacol.

[16] Berridge MJ (1997). Elementary and global aspects of calcium signalling. J Physiol.

[17] Fan X, Ma J, Wan W, Zhang P, Wang C, Wu L (2011). Increased intracellular calcium concentration causes electrical turbulence in guinea pig ventricular myocytes. Sci China Life Sci.

[18] de Diego C, Pai RK, Chen F, Xie LH, De Leeuw J, Weiss JN, Valderrabano M (2008). Electrophysiological consequences of acute regional ischemia/reperfusion in neonatal rat ventricular myocyte monolayers. Circulation.

[19] Casini S, Verkerk AO, van Borren MM, van Ginneken AC, Veldkamp MW, de Bakker JM, Tan HL (2009). Intracellular calcium modulation of voltage-gated sodium channels in ventricular myocytes. Cardiovasc Res.

[20] Rocchetti M, Besana A, Mostacciuolo G, Ferrari P, Micheletti R, Zaza A (2003). Diverse toxicity associated with cardiac Na+/K+ pump inhibition: evaluation of electrophysiological mechanisms. J Pharmacol Exp Ther.

[21] Shutt RH, Ferrier GR, Howlett SE (2006). Increases in diastolic [Ca2+] can contribute to positive inotropy in guinea pig ventricular myocytes in the absence of changes in amplitudes of Ca2+ transients. Am J Physiol Heart Circ Physiol.

[22] Walton RD, Martinez ME, Bishop MJ, Hocini M, Haissaguerre M, Plank G, Bernus O, Vigmond EJ (2014). Influence of the Purkinje-muscle junction on transmural repolarization heterogeneity. Cardiovasc Res.

[23] Sicouri S, Belardinelli L, Antzelevitch C (2013). Antiarrhythmic effects of the highly selective late sodium channel current blocker GS-458967. Heart Rhythm.

[24] Zhao YT, Valdivia CR, Gurrola GB, Hernandez JJ, Valdivia HH (2015). Arrhythmogenic mechanisms in ryanodine receptor channelopathies. Sci China Life Sci.

[25] Asakura K, Cha CY, Yamaoka H, Horikawa Y, Memida H, Powell T, Amano A, Noma A (2014). EAD and DAD mechanisms analyzed by developing a new human ventricular cell model. Prog Biophys Mol Biol.

[26] Dorian P, Newman D (2000). Rate dependence of the effect of antiarrhythmic drugs delaying cardiac repolarization: an overview. Europace.

[27] Grom A, Faber TS, Brunner M, Bode C, Zehender M (2005). Delayed adaptation of ventricular repolarization after sudden changes in heart rate due to conversion of atrial fibrillation. A potential risk factor for proarrhythmia?. Europace.

[28] Catterall WA (1988). Structure and function of voltage-sensitive ion channels. Science.

[29] Catterall WA (2000). From ionic currents to molecular mechanisms: the structure and function of voltage-gated sodium channels. Neuron.

[30] Sawanobori T, Hirano Y, Hiraoka M (1987). Aconitine-induced delayed afterdepolarization in frog atrium and guinea pig papillary muscles in the presence of low concentrations of Ca2+. Jpn J Physiol.

[31] Watano T, Harada Y, Harada K, Nishimura N (1999). Effect of Na+/Ca2+ exchange inhibitor, KB-R7943 on ouabain-induced arrhythmias in guinea-pigs. Br J Pharmacol.

[32] Zhao Z, Yin Y, Wu H, Jiang M, Lou J, Bai G, Luo G (2013). Arctigenin, a potential anti-arrhythmic agent, inhibits aconitine-induced arrhythmia by regulating multi-ion channels. Cell Physiol Biochem.

[33] Zhou YH, Piao XM, Liu X, Liang HH, Wang LM, Xiong XH, Wang L, Lu YJ, Shan HL (2013). Arrhythmogenesis toxicity of aconitine is related to intracellular ca(2+) signals. Int J Med Sci.

[34] Ren Z, Ma J, Zhang P, Luo A, Zhang S, Kong L, Qian C (2012). The effect of ligustrazine on L-type calcium current, calcium transient and contractility in rabbit ventricular myocytes. J Ethnopharmacol.

